# Investigation of Timing Behavior and Jitter in a Smart Inertial Sensor Debugging Architecture

**DOI:** 10.3390/s21144675

**Published:** 2021-07-08

**Authors:** Daniel Gis, Nils Büscher, Christian Haubelt

**Affiliations:** Institute of Applied Microelectronics and Computer Engineering, Faculty of Computer Science and Electrical Engineering, University of Rostock, 18059 Rostock, Germany; nils.buescher2@uni-rostock.de (N.B.); christian.haubelt@uni-rostock.de (C.H.)

**Keywords:** inertial sensors, evaluation, hardware-in-the-loop, repeatability, reproducibility, smart sensor

## Abstract

Due to upcoming higher integration levels of microprocessors, the market of inertial sensors has changed in the last few years. Smart inertial sensors are becoming more and more important. This type of sensor offers the benefit of implementing sensor-processing tasks directly on the sensor hardware. The software development on such sensors is quite challenging. In this article, we propose an approach for using prerecorded sensor data during the development process to test and evaluate the functionality and timing of the sensor firmware in a repeatable and reproducible way on the actual hardware. Our proposed Sensor-in-the-Loop architecture enables the developer to inject sensor data during the debugging process directly into the sensor hardware in real time. As the timing becomes more critical in future smart sensor applications, we investigate the timing behavior of our approach with respect to timing and jitter. The implemented approach can inject data of three 3-DOF sensors at 1.6 kHz. Furthermore, the jitter shown in our proposed sampling method is at least three times lower than using real sensor data. To prove the statistical significance of our experiments, we use a Gage R&R analysis, extended by the assessment of confidence intervals of our data.

## 1. Introduction

In recent years, inertial sensors have become more common for a wide range of applications implemented on a multitude of different devices. The applications range from gesture recognition [1] to step detection [2] to autonomous driving or Unmanned Aerial Vehicles (UAVs) [3,4].

Modern inertial sensors are often so-called smart sensors. This type of sensor consists of the actual sensor device and an additional processing unit. Using this unit, smart sensors are capable of preprocessing captured data directly on the sensor before they are used and processed by the system application processor.

In a big variety of applications, especially in safety critical systems, it is crucial that the whole system, including the inertial sensors, can be evaluated for all possible aspects. Evaluating systems regarding their physical aspects can be quite challenging [5], depending on the used test environment. To obtain meaningful results, it is necessary to inspect the whole system including the preprocessing or filtering part on the inertial sensors. For this sensor system evaluation there are mainly two state-of-the-art methods used. The first method is using simulation models for the system evaluation. There are a lot of implementations, using this simulation approach. In such systems it is popular to only simulate specific parts of the whole system, e.g., just the data path or instruction set. The simulation of a whole system takes a lot of simulation time and processing performance, which makes it not suitable for real-time simulations of whole sensor systems. The second method uses hardware prototypes of the sensors, which are often mounted on a prototype printed circuit board. This makes it easy to build a rapid prototype for testing smart sensors in a whole sensor system environment. Using the hardware prototype, enables the developer to test all aspects of the smart sensor. This includes extra functional properties such as power consumption or heat distribution. The big disadvantage of this method is the missing reproducibility. With the current methods using hardware prototypes it is not possible to repeat a test with the same sensor data. A further classification and description of these approaches can be found in Section 2.

Both methods of sensor system evaluation, have their advantages, but also their drawbacks. To overcome the drawbacks, we proposed a novel approach in [6] which can be compared to a Hardware-in-the-Loop (HiL) method [7]. This approach aims to combine all positive aspects of the previously described methods and uses real hardware in a prototype or on an evaluation board. It can use sensor data prerecorded from the very same hardware as an input from the sensor. By re-injecting previously captured sensor data it can create reproducible system test results because the sensor processes the injected data such as real sensor data. Hence, our approach supports the development, debugging, and profiling of the behavior of the physical sensor in a repeatable and reproducible way. This enables the developer to observe functional and extra functional system properties such as system resources, run time or power consumption in real time on the running target sensor. The proposed method is proven capable of dealing with three 3-dimentional inertial sensors with a sampling frequency of up to 1600 Hz. This makes it very useful for a broad variety of systems and application.

Besides the correct functionality, the timing behavior of such systems becomes more and more critical. In particular, the jitter in the time series of the sensor data has a significant influence on the quality of the functional properties [8]. Since in [6] we only considered the functionality of the system and did a first performance estimation, in this article we will do a proper analysis of the timing behavior. Furthermore, we will investigate the influence of the jitter on said system. In both cases, timing and jitter analysis, we will focus on the replay or injection phase of our approach. The behavior in this phase is very critical, because sampling real sensor data and using injected data must be as similar as possible in timing behavior. Therefore, we did a further investigation on the timing behavior of our approach considering both debug and optimized production firmware. An extensive analysis of the timing jitter comparing real data sampling and sampling using the Sensor-in-the-Loop (SiL) approach has been done. In our jitter analysis in Section 6, we investigated Cycle-to-Cycle Jitter (Jcc), peak-to-peak Cycle-to-Cycle Jitter (Jpp) and Periodic Jitter (Jper). The jitter using our approach is at least three times lower than using the actual hardware sensors. To prove the statistical significance of our results, we conducted an extensive statistical analysis using Gage Repeatability and Reproducibility (Gage R&R) analysis extended by the determination of the confidence interval of the results.

In the following sections, we will describe our approach for debugging smart inertial sensors in a repeatable and reproducible way. This approach enables the sensor firmware developer to inject prerecorded or artificially created sensor data and stimulate the smart sensor firmware running on the target system. As a result, the developer can gain higher confidence in the functionality and time behavior of the firmware. Hence, our proposed method closes a gap between simulation-based and rapid hardware prototype debugging approaches. To increase the applicability, the injection process must support multiple sensors simultaneously and provide direct feedback from the on-sensor computations. In our experiments, we systematically study these aspects and show the viability of our approach.

The work at hand is structured as follows:

At first, we will discuss the related work in Section 2. Afterwards, in Section 3, a general overview of smart sensors is given. The concept used for our Sensor-in-the-Loop architecture is explained in Section 4, with the prototypical implementation described in Section 4.4. Subsequently, the prototypical implementation is analyzed for its timing behavior in Section 5. The timing analysis is enhanced with an analysis of the jitter in Section 6. The analysis results are afterwards discussed in Section 7. To ensure that our results are statistically reliable the statistical significance of the results is explained in Section 8. Finally, the results and findings of the work at hand are discussed in Section 9.

## 2. Related Work

The evaluation and simulation of inertial sensors and inertial sensor systems are common tasks in the development of a system using said sensors. Therefore, numerous software suites and approaches exist for these tasks. One example is dSPACE which offers a Hardware-in-the-Loop *Sensor Simulation PC*. It allows for a deterministic simulation of the physical models of a sensor in real time [9]. The algorithms can be evaluated in conjunction with the sensor data. However, the *Sensor Simulation PC* is primarily targeted at autonomous driving. Therefore, it provides simulation data for camera sensors, LIDAR, and RADAR. In contrast to our work, the *Sensor Simulation PC* platform does not inject the sensor data back into the sensor to simulate that the sensor device is providing the data. Therefore, an evaluation of the behavior on the real hardware is not entirely possible.

The *Sensor Fusion and Tracking Toolbox* from *MatLab* [10] or the GNSS-INS-SIM [11] are other toolboxes which provide the possibility to simulate inertial sensor data to develop and evaluate sensor fusion algorithms. Both toolboxes provide a high-level software suite and tools. The *Sensor Fusion and Tracking Toolbox* from *MatLab* already contains multiple different predefined algorithms for object detection, position tracking, or situation recognition. One can use either recorded or completely synthetic data. Both toolboxes are completely simulation-based and work at a high abstraction level. This makes it impossible to determine the behavior on real hardware or the hardware requirements such as power consumption, memory usage or the ability to run in real time.

The state-of-the-art approach to evaluate an inertial sensor system is using a simulation model with a so-called virtual prototype. This VP not only simulates the functional behavior of system but also its extra functional properties such as timing, power consumption, memory requirements, etc., i.e., it simulates the used hardware [12]. For example, in [13], the authors present a RISC-V processor module which includes a sensor submodule. There are other free virtual prototypes available for a wide range of architectures [14]. This approach does not need the actual hardware to evaluate the developed fusion algorithms but still is quite accurate regarding the behavior of the hardware. In the work [12], the inertial sensor is simulated using the physical characteristics that can be extracted from its datasheet. However, to accurately simulate complex hardware systems in every detail can become very computational expensive, and it is often not possible to simulate the hardware in real time. Additionally, the accuracy of the simulation of the hardware is limited. It is typically not possible to simulate all physical details of the hardware because they are either not documented or unknown. One example is a jitter that can come from either the inertial sensor measurement process or the communication between elements. In contrast, our work needs the actual hardware, but it can still produce repeatable results using prerecorded data. Therefore, all properties of the whole system can be considered.

Using a prototype- or application board containing the real sensors is another state-of-the-art method to evaluate the inertial sensors [15,16]. This allows for an evaluation of the actual hardware in conjunction with the software. All effects depending on the physical properties of the hardware can be seen using this approach. However, to generate reproducible data input for different evaluation runs is challenging or even impossible using this method. To use robots which can perform deterministic movements is a way to make measurements repeatable and can be a possibility for some use cases. However, due to hardware limitations, robots are not able to simulate real human movements or very complex movements in detail.

The work at hand builds upon the idea of using the actual hardware. However, it enhances this method by adding the ability to not only capture life data but also to play back previously recorded data or generated data on the real sensor. This enables reproducible results without the need for robots or additional hardware to reproduce movements. Table 1 lists the technologies of all presented approaches in this section. It shows the relevant properties of all technologies in comparison to our Sensor-in-the-Loop approach.

One important aspect of the evaluation done in the work at hand is the influence of the injection process of recorded data on the behavior of the hardware and software. In these processes, timing jitter will have a significant influence on the functionality and the performance. This jitter can be caused by the communication interface, processing of the data or scheduling of the Microcontroller (µC). In [8] it was shown that the jitter can have a significant impact on the result. Other authors have investigated the influence of sampling jitter on control systems. Some of these works even propose methods to handle sampling jitter in such systems. In [17], the author showed the effect of sampling jitter on a servo motor controller. He proposed an approach to stabilize the control system under the influence on the jitter. The authors in [18] showed the effect of sampling jitter on an PID controller implemented in a real-time system on a µC. The conclusion of this work is that sampling error can lead to instability of such systems. Furthermore, they present a practical approach to deal with sampling error on their test system. In [19], the authors write that jitter can influence the control performance in control systems or in the worst case lead to the instability of the system.

## 3. Smart Sensors

Smart sensors become more and more important in a wide range of sensing applications. There is already a wide variety of different kinds of smart sensors available on the market.

Using a conventional sensor, the sensor output is usually represented by a voltage level on the output pins. This voltage level can be sampled using an Analog to Digital Converter (ADC) which is connected to some kind of host processor or µC. Other types of sensors already integrate the ADC so that the host can read the sensor data using a digital interface such as the serial peripheral interface (SPI) or an Inter-Integrated Circuit (I^2^C).

In addition to the actual Micro-Electro-Mechanical Systems (MEMS) sensing element and the Analog to Digital Converter, a smart sensor contains some kind of processing unit and memory. Using this Sensor-Processing Unit (SPU), the developer can perform calculations and data manipulations directly on the sensor before communicating them to the host. Usually, the SPU can be programmed by a sensor firmware so that the sensor functionality can be changed any time.

**Figure 1:**
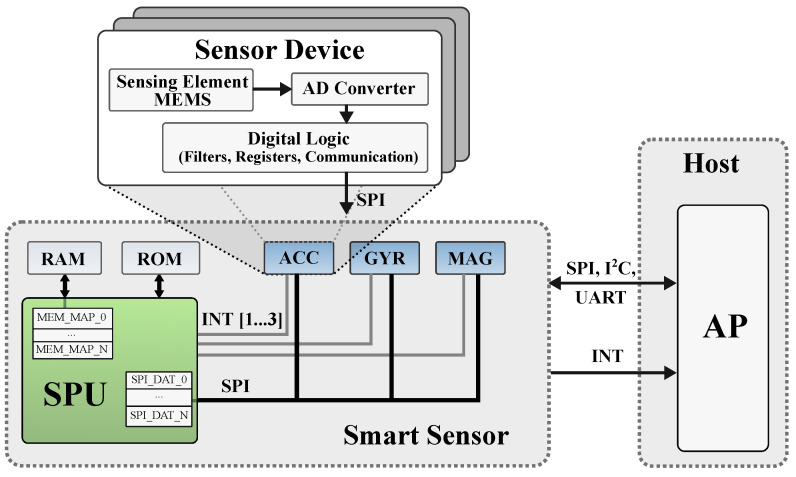
Typical structure of a smart sensor.

Depicted in Figure 1 is a typical smart sensor that consists of three separate components. One or more sensor devices, the SPU and additional memory.

The sensor devices are depicted in blue. They are not only limited to inertial sensors, such as accelerometers and gyroscopes, but may implement any type of sensor, e.g., magnetometer, temperature sensors or pressure sensors. Usually, we do not work with optical sensors with very high data rates such as high-resolution cameras. The sensor itself can be divided into three subcomponents as depicted in Figure 1. These components are the actual sensing element, commonly manufactured in MEMS technology, the ADC to convert the analog signal from the sensor into a digital signal and the digital logic that allows a preprocessing of the data, e.g., a low-pass or averaging filtering. The preprocessed data are then stored into registers that can be read by the SPU via a communication interface.

There are multiple different interfaces for inter-circuit communication between the sensor devices and the SPU in a System in Package (SiP). Most commonly used are the SPI or I^2^C. Often, sensors are also connected with an interrupt line to the SPU for a low latency notification of events. The interrupts are used, for example, to signal the arrival of new data and for certain predefined events such as a detected step, the exceeding of a threshold level or a significant motion.

The SPU within the smart sensor is the actual smart part of the sensor. It enables the application developer to combine the data from all available sensors at one location and process the data directly on the sensor hardware. The processing can include the combination of multiple sensors to a so-called virtual sensor, a compression of the data to reduce the communication overhead to the Application Processor (AP), a filtering of the data, or the detection of gestures or steps. Typically, the SPU in a smart sensor is an energy efficient µC such as an ARM Cortex M0+ e.g., in the BMF055 by BST [20] or an application specific proprietary processing unit as used in [21]. Generally, the SPU is accompanied by an additional memory unit that is connected via a memory interface or a standard communication interface. The Read Only Memory (ROM) contains the actual program code that is executed on the SPU and constant values used by the program. The RAM is used for intermediate results, the function stack, or acts as a buffer for the communication with the AP.

Smart sensors can be classified into three different integration levels. A detailed description of the integration levels System On Chip (SoC), SiP and System on Module (SoM) can be found in [6]. Our proposed SiL concept is suitable for all three kinds of smart sensor implementations and combinations of them.

## 4. Sensor-in-the-Loop

When designing software for smart sensors, there is a need for testing and verification methodologies. In the work at hand, we focus on our previously published approach called SiL. In the following, the basics of this approach are introduced to thoroughly analyze its timing behavior in later chapters. The SiL architecture consists of three parts that are connected via a common interface. The separation into three parts allows for a better maintainability and interchangeability of the components. The three parts will be described bottom-up with the sensor firmware at the lowest level, followed by the debugger interface and the Graphical User Interface (GUI).

### 4.1. Sensor Firmware

The SPU or the µC that supports the proposed SiL approach must handle the recording and the injection of the sensor data from the host PC. Therefore, the firmware must be adapted and either implement a library or a collection of functions to add support for the named SiL functionalities. Optimally, these functions should look and behave similar to the standard Application Programming Interface (API) functions of the smart sensor to read and receive data from the connected sensors using a common communication interface such as SPI or I^2^C. The sensor firmware therefore works as the interface between the sensor device, the SPU and the GUI. To have a similar behavior between the injected sensor data and real recorded sensor data, the modified firmware functions must show the same timing behavior for both data paths.

### 4.2. Communication Interface

The communication interface establishes the connection between the SPU of the sensor and the host computer running the control and view interface. The communication interface can be implemented using a variety of different physical interfaces and protocols. However, depending on the number of sensors and the used sampling rate, following criteria must be met:

- Data order
- Reliability
- Throughput
- Latency

The correct order of the sensor data packages is mandatory. The data must be injected and read in the same order as they have been recorded. Additionally, the communication interface must be reliable to ensure that no data packages are lost, and the sensor processes the injected data identically for every run. The communication interface must provide a higher throughput than the combined data of all sensors requires, since it can be necessary to send additional control data or calculation results. The minimal throughput $R_{min}$ can be estimated using Equation (1).

(1) $$R_{min}=\sum_{n=1}^{N}\left(fs_{n}\cdot \left(bw_{n}\cdot ac_{n}+P_{OH}+ACK_{OH}\right)\right)+CI_{OH}$$

The required throughput for each individual sensor consists of the sensor sampling frequency $fs_{n}$, the bit-width $bw_{n}$ of the data and the number of signals per sensor $ac_{n}$. For each sensor, there is an additional acknowledgment overhead $ACK_{OH}$ and protocol overhead $P_{OH}$ that are needed for the used communication protocol. The required individual throughputs are summed up for all *N* sensors. To the sum of the throughput of all sensors comes an additional throughput $CI_{OH}$ that is required by the communication interface and depends on the chosen interface and can vary from no overhead at all to a multiple of the actual sensor data.

The Equation (2) shows an example for a system which consists of a 3-dimensional 16 Bit accelerometer sampled with 2 kHz and a 16 Bit temperature sensor at 1 Hz. Additionally, to the sensor data, the packet overhead $P_{OH}$ is 2 Byte and the acknowledgment packet for each sample has a length of 3 Byte. Assuming that the communication interface overhead $CI_{OH}$ is 0 Byte/s the overall required throughput results in 22,007 Byte/s.

(2) $$\begin{array}{cc} R_{min} & =(2000\cdot (2\cdot 3+2+3))+(1\cdot (2\cdot 1+2+3)+0\\ & =22,007\;\;\mathrm{Byte}/s \end{array}$$

The final important criterion for the communication interface is the latency. It must be small enough to transfer the sensor data of all sensors in real time. The latency highly depends on the output data rate or sampling frequency of the sensors. For those criteria, the sensor with the highest output data rate is the one determining the overall latency.

All named criteria must be fulfilled; however, it must not all be done in the communication interface. It can still be possible to ensure some requirements on a higher protocol level.

### 4.3. View and Control / Graphical User Interface

The View and Control Interface (VCI) is the third part of the SiL architecture. With the help of the VCI it is possible to record the sensor data from the sensor that supports SiL in the desired data rate. The recorded data can be re-injected into the sensor to verify different versions of the sensor firmware to make sure it works or to see if an improvement was successful in a reproducible manner. The VCI should also allow the engineer to manipulate the recorded data to e.g., test the firmware for different error scenarios. Another important function of the VCI is to visualize the recorded data and calculation results or detected events from the sensors. Seeing the data from the sensor in real time helps the engineer to develop, improve, and evaluate the sensor firmware in an easy and targeted way.

### 4.4. Example Implementation on BMF055

The three components proposed in Section 4 have been implemented in a prototype as a proof of concept to show the usability of the proposed SiL architecture. This section will describe the implementations of the three components in the same order as in Section 4.

**Figure 2:**
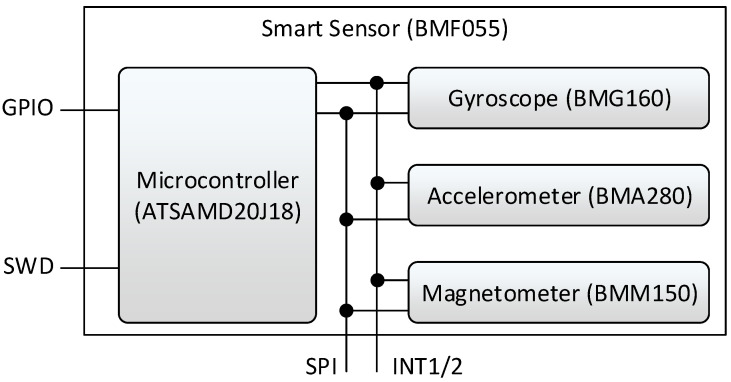
BMF055 components.

The firmware of the SiL prototype is implemented on a BMF055 programmable smart sensor from Bosch Sensortec [20]. This smart sensor is implemented as a System in Package. Hence, the sensor consists of different dice which are all connected in the same package. The BMF055 contains three sensors, a 16-bit triaxial gyroscope [22], a triaxial 14-bit accelerometer [23] and a triaxial geomagnetic sensor [24]. The µC used in the BMF055 is the 32 bit Cortex M0+ microcontroller [25]. The components of the BMF055 are depicted in Figure 2.

#### 4.4.1. Microcontroller Firmware Implementation

**Figure 3:**
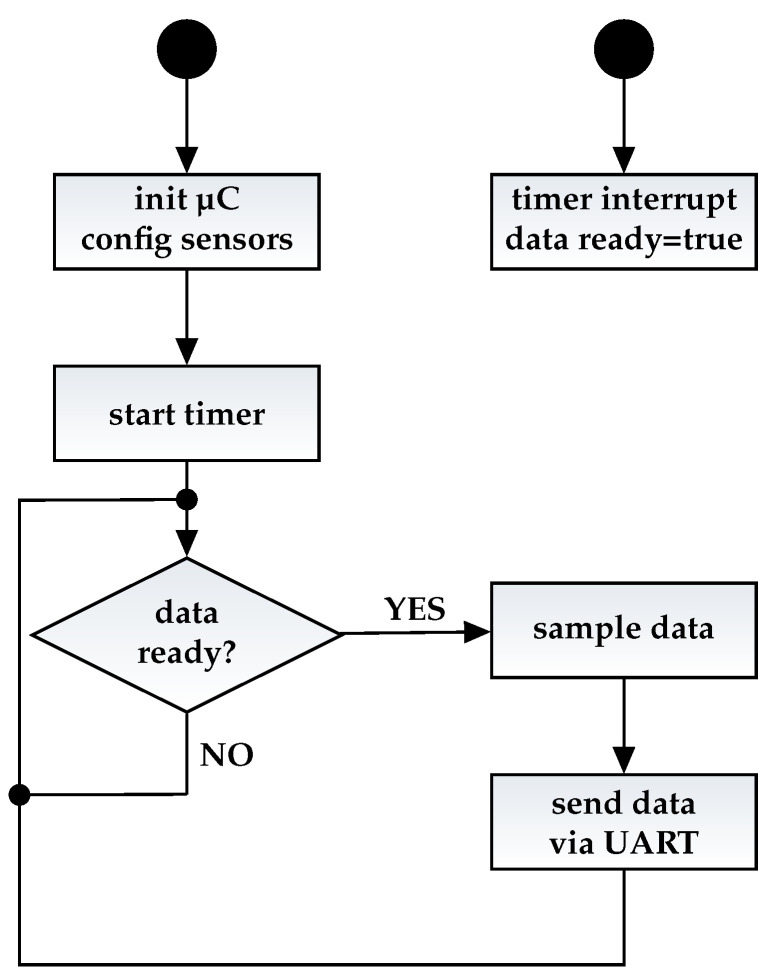
Flow chart of the example firmware implementation.

The SiL demonstration application has been implemented using the example firmware implementation of the BMF055 called “Data Stream”. This example shows how to sample data from all three sensors in a straightforward manner. The firmware samples the data from the sensors with an individual configurable data rate and sends it to the host using Universal Asynchronous Receiver Transmitter (UART) interface of the µC. The example firmware has been extended to implement the SiL library and provide the necessary SiL functionality. The flow chart of the firmware can be seen in Figure 3.

The implemented SiL C-library offers the functionality to send recorded data from the sensors to the GUI on the host computer. On the host, the recorded values can be visualized and processed. The main functionality of the C-library is the possibility to receive data from the host computer and process it in the same way as data that has been sent by the sensors.

An important factor, especially in an embedded system, is the memory footprint of the firmware. Obviously, the added functionality for the SiL features adds to the memory footprint. To ensure that added features do not have a large impact on the memory footprint, we analyzed the memory size of the firmware on the µC.

In [6] we show that the memory overhead of the SiL is negligible, the example implementation uses only around 0.63% of the flash memory.

#### 4.4.2. J-Link RTT Interface

The communication between the firmware and the host is realized using the Segger Real-Time Transfer (RTT) interface. This interface offers the ability for real-time communication and contains other features that are useful for running the prototypical implementation. The hardware used is the Segger J-Link Base hardware debugger [26] which integrates the transfer protocol and allows a developer to debug the sensor firmware. This lite version has a limited transfer speed but is sufficient to inject the sensor data back into the sensor. If higher data rates than the ones used in the experiments in Section 5 are required, it is possible to use the Segger J-Link Pro, PLUS or ULTRA+ versions. Using the J-Link hardware debugger has the benefit that no additional hardware is needed for the implementation of the SiL features. More information about the used RTT interface can be found on the Segger website [27].

**Figure 4:**
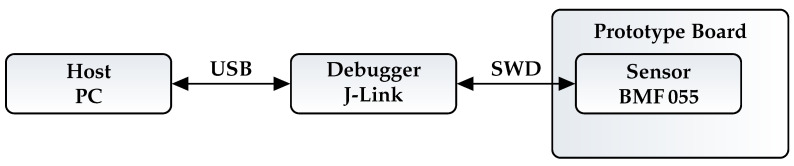
Physical connections used on the setup.

The debugger is connected to the host via a USB cable. The actual smart sensor is held by a BMF055 shuttle board which is placed on a breakout board provided by Bosch Sensortec. The standard 20 pin connector from the breakout board is connected to the J-Link debugger via the Serial Wire Debug Interface (SWD). Figure 4 shows an overview of the physical connection.

#### 4.4.3. SiL View and Control

The GUI part of the SiL architecture was implemented as an eclipse plugin that provides all features needed to evaluate and control the prototype. Using an eclipse plugin allows for an easy integration into an already established development environment which provides all tools to develop and debug the smart sensor from a commonly used open source Integrated Development Environment (IDE).

**Figure 5:**
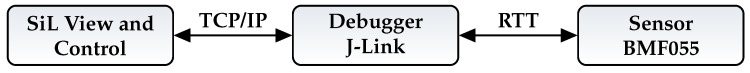
Communication protocols used on the setup.

The communication with the target is via a Transmission Control Protocol (TCP) socket offered by the J-Link. The data flow of the SiL packages for the sensor application is controlled using the standard socket implementation in Java. The debugger uses the RTT interface for the communication with the sensor after receiving the data from the socket. The used connection protocols are depicted in Figure 5.

To use the SiL features of the smart sensor, first the debug process must be started using the standard debug target option of eclipse. Once the debugging process has started, it is possible to connect to the smart sensor using the Sensor View plugin.

**Figure 6:**
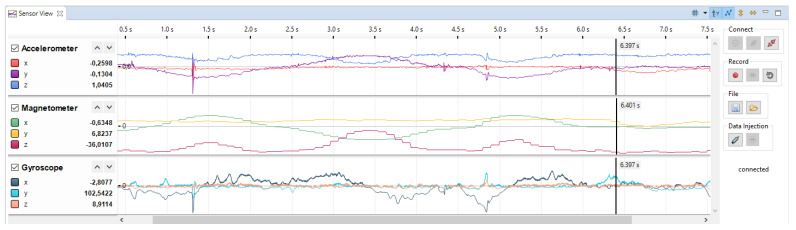
Sensor View in Eclipse environment.

Currently the eclipse plugin provides three features. The first functionality is to fetch the data from the sensor and display it live in the GUI. It is possible to step through the code of the firmware and still see the sensor data. The data shown in GUI will be in sync with the time base of the sensor. The second feature is the recording of the sensor data. As soon as the data from the sensor is fetched, it is possible to save the complete data set or a subset of it in a Comma Separated Values (CSV) file. It is possible to use one CSV file for all sensors or a separate file for each of the sensors. The third functionality is the injection of the previously recorded data back into the sensors. Therefore, it is possible to load and display the recorded data and send it back to the sensor. It is possible to simultaneously inject and record data. To help the developer, the GUI displays the currently injected data and the new data received by the sensor synchronized side by side. For example, the sensor can calculate the quaternion representation of the sensor orientation using the injected data from gyroscope and accelerometer. Figure 6 depicts the Sensor View which shows the recording of sensor data from a three-dimensional accelerometer, gyroscope, and a three-dimensional magnetometer.

The Sensor View can handle signals with different sampling rates at the same time. The data from the accelerometer displayed in Figure 6 is sampled with 800 Hz. The data from the magnetometer is sampled with 100 Hz and the gyroscope shows a sampling rate of 1.6 kHz. The controls for the GUI are located at the right, grouped by their functionality. The first group contains the controls to connect to the sensor as well as the selection of the configuration file for the whole session. The second group handles the recording of the data from the sensor. It also offers the possibility to reset the captured data. The controls to save fetched sensor data or load previously recorded data are organized in group 3. The last group contains the controls to start and stop the injection of the loaded sensor data back into the sensor. Using the checkboxes left to the signals, one can choose, which signals should be injected into the sensor on the next run.

#### 4.4.4. Data Buffers

**Figure 7:**
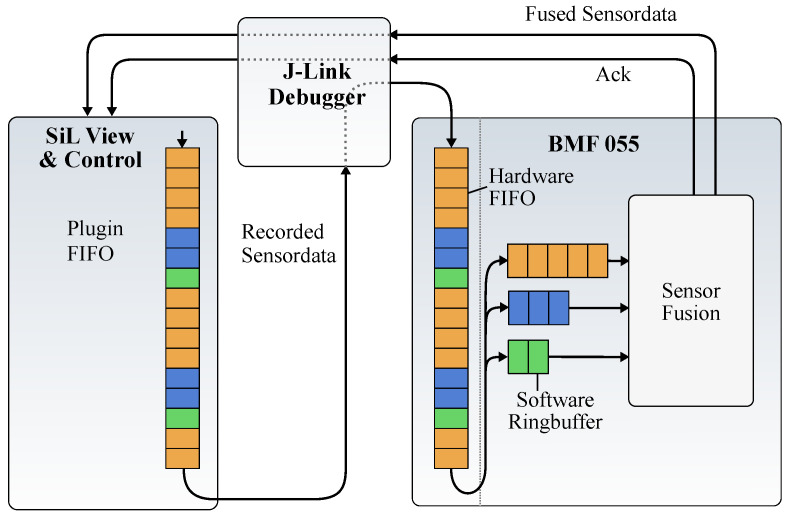
Buffer structure of the SIL Setup.

The SiL architecture should be usable with different sensors and different connection interfaces. Using a different communication interface can mean that the bandwidth as well as the latency differ from our prototype implementation. To handle the possibility of lower bandwidth or a higher latency, we implemented an additional buffer structure in the SiL View and Control as well as the sensor firmware which is shown in Figure 7.

The buffer of the eclipse plugin is a typical First In First Out (FIFO) buffer that holds an individual amount of sensor samples for each sensor. The length of this FIFO is configurable and depends on the latency of the used communication interface. It is very common that the different sensors of a smart sensor or sensor hub are sampled with a different frequency. Therefore, the individual sensors require different buffer sizes. In the eclipse plugin, this is handled by filling the FIFO with a different amount of data for each sensor. The number of samples added for each sensor is calculated with Equation (3).

(3) $$B_{SIZE_{N}}=\max\left(2,\frac{f_{i}}{f_{ges}}\cdot B_{SIZE_{MAX}}-\left(\left(N-1\right)\cdot 2\right)\right)$$

(4) $$f_{ges}=\sum_{n=1}^{N}f_{n}$$

In Equation (3) $f_{ges}$ is the sum of all individual frequency from the sensors and is calculated in Equation (4). The frequency $f_{i}$ is the frequency of the sensor for which the buffer size should be calculated. $B_{SIZE_{MAX}}$ is the overall size of the sensors, *N* is the number of used sensors. Each sensor is given a minimum buffer size of at least 2 samples in the FIFO independent of the actual sampling rate.

Inside the firmware of the sensor, the incoming hardware FIFO buffers the incoming data. These data are split into individual buffers for each sensor. For performance and reliability reasons these buffers are implemented as ring-buffers.

For the fetching of the data from the sensor and their visualization in the GUI, no additional buffers are used because this direction is not time critical. All sent sensors samples contain a time stamp to reconstruct the timing behavior after the plugin received the data.

## 5. Timing Experiments

After the SiL features described in Section 4.4 have been implemented, they were evaluated for a correct behavior. The testing was conducted using the firmware implementation of the stream example depicted in Figure 3. In [6], we did a first evaluation of the correct behavior of our approach regarding timing. Since timing and especially jitter are very critical parameters in sensor systems, we will do an extensive analysis of both parameters in the following two sections. In this section, we will focus on the actual timing followed by the investigation of the sampling jitter in the next section. The tests included measurements for the time needed to sample sensor data via the SiL interface. Finally, we compare the results against the standard driver implementation.

There exists multiple different methods to measure the time in a bare metal C program on a µC. The easiest method is to use a system timer or a General-Purpose Timer. These timers count the number of clock cycles since they are started. Starting the timer before the sampling and stopping right after allows calculation of the actual time taken by the sampling method. The actual time can be calculated by multiplying the cycle count with the known clock frequency of the µC. This method is easy to implement because it does not need any additional hardware and can result in an accurate measurement of the timing behavior. To make sure that the results are valid, it must be ensured that the timer clock does not change during the whole measurement.

Another method to measure the timing behavior is the usage of an external device such as an oscilloscope or logic analyzer using a General-Purpose Input Output (GPIO) pin. At the start of the measured period the GPIO is set to *high* and reset to *low* at the end of the measurement period. The length of the time period can then be measured by observing the voltage level of the GPIO pin by an oscilloscope or logic analyzer.

For the timing experiments, we used the second method because it is independent of the used sensor firmware and the accuracy of the measured time period is not influenced by the sensors clock frequency but by the accuracy of the used logic analyzer. Generally, the clocks used in oscilloscopes or logic analyzers are much more accurate than the ones used in small µC.

The results of the timing measurement for the -O0 optimization level and for the -O3 optimization level are shown in Section 7. The measurements were done using the Saleae Logic Pro 8 [28] USB logic analyzer, which is an 8 channel 500 MS/s (500 million samples per second) logic analyzer. The sampling rate is high enough to obtain significant and accurate results for the desired measurements.

**Figure 8:**
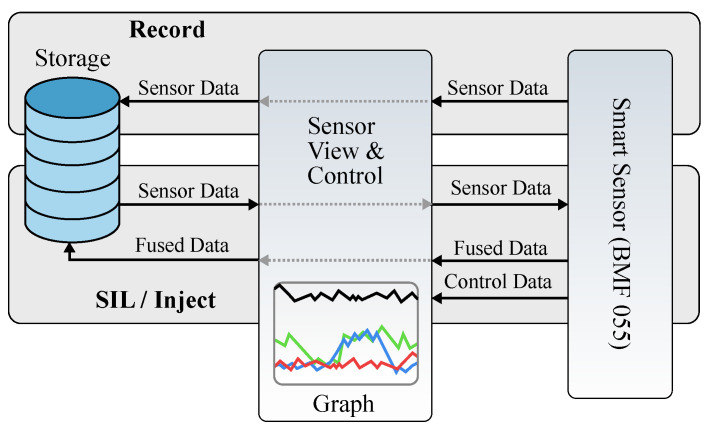
Data Flow of the SIL Setup.

For the experiment, the timing behavior when sampling the real sensor data and the timing behavior when sampling the data via SiL interface have been measured and compared. The sampling of the real physical sensor data was done using the SPI interface operating with a 10 MHz bus clock. The sampling via the SiL interface was done using a previously recorded CSV file. The recording part is shown in Figure 8.

The figure depicts the data flow of the whole SiL concept. The record and store part is depicted in red, the injection is depicted in blue. The timing behavior for sampling the data from the real sensors is used as reference to compare the timing behavior of the SiL injection against.

As shown in Tables 3 and 4, three different scenarios were measured. All tests were done with a data set with a length of 10 s. In the first test, the data of the accelerometer was injected with a sampling rate of 1600 Hz. For the second and the third test, the data of all three physical sensors, gyroscope, magnetometer and accelerometer have been injected. In the second test, all three sensors are configured with an identical sampling rate of 1600 Hz. In the third test, the sampling rate of the sensors was varied, to evaluate the unbalanced filling of the buffers. The accelerometer was sampled at 800 Hz, the gyroscope at 1600 Hz and the magnetometer at 100 Hz.

As mentioned before, we used a logic analyzer to measure the timing behavior using the level changes of a GPIO pin. Switching the GPIO pin itself to *high* or *low* also takes time, both as instruction in the firmware and physically to switch the voltage level. The time it takes for toggling the pin without any other instructions has been measured beforehand and can be considered in the other measurements to obtain absolute timing values without this overhead.

It was also measured how long it takes to record the fetched sensor data from the µC and send it to the host via the SiL functionality. This time cannot be compared to any functionality of a sensor without a SiL interface, but it is useful to know how much the record functionality effects the timing behavior of the firmware.

## 6. Jitter

To further investigate the timing behavior of our implemented Sensor-in-the-Loop approach, we studied the jitter of different methods to sample sensor data. In this section, we will give a brief overview over the different kinds of jitters we have measured in our setup. We will describe the experimental setup and the measured methods of sampling sensor data including sampling real sensor data and SiL sensor data.

**Figure 9:**
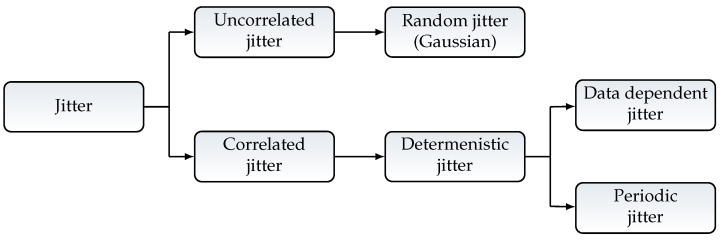
Types of jitter.

In current state of literature, jitter is described as a deviation in time from the real clock edge or periodicity in a periodic signal [29,30,31]. Usually, the overall jitter is composed of two categories, random jitter and deterministic jitter [31]. Figure 9 shows an overview of the different kinds of jitters and their relations.

Random jitter is present in every system and is a broadband stochastic Gaussian Process that is described as unbound. Sometimes it is also described as intrinsic noise. Random jitter can have a big contribution to the overall jitter in a System [30].

Deterministic jitter always has a specific cause, it can be periodic and is often narrowband. The periodicity can be seen in one or more frequencies of the signal [29]. There are mainly two types of deterministic jitter. The first type is called Periodic Jitter, it can be caused by a switching power supply, a close clock signal, or even by the implementation of the system clock itself. The second type of deterministic jitter is data-dependent jitter. The detection of this type of jitter is not that easy, because it depends on the system, the implemented functionality, and other factors [29]. This can lead to a misinterpretation of data-dependent jitter as uncorrelated or random jitter.

Furthermore, jitter can be categorized in correlated and uncorrelated. Correlated jitter is always deterministic and correlated with some kind of source. It can be periodic or aperiodic. Uncorrelated jitter e.g., Random jitter is not statistically connected to any kind of defined source. In the case that there is an overlay of different correlated jitters, they can appear as uncorrelated without a deeper analysis.

There are different state-of-the-art jitter measurements described in the literature [29,30,31]. For the results shown later in this article, three types of jitter measurements were used.

### 6.1. Cycle-to-Cycle-Jitter

**Figure 10:**
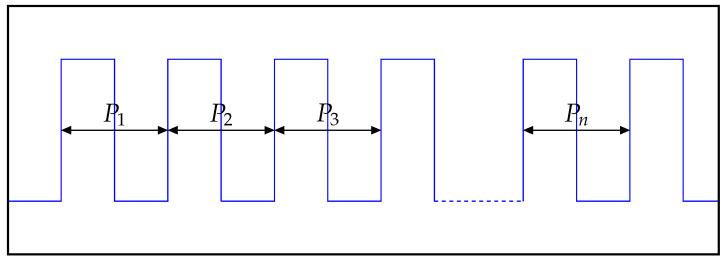
Cycle-to-Cycle-Jitter.

Jcc can be described as the difference in time between two adjacent and consecutive clock cycles. In Figure 10 one can see a signal pictured by several of its periods. All depicted periods $P_{1}$ to $P_{n}$ are not ideal, what means their length is different in time compared to the desired length for one period defined for that signal.

Using Equation (5), the Cycle-to-Cycle-Jitter can be calculated for the measured signal. Jcc is a list of values which contains the separate Jcc values for each period of the signal. The symbols $P_{1}$ to $P_{n}$ represent the individual periods of the considered signal.

(5) $$Jcc=|P_{2}-P_{1}\left|,\right|P_{3}-P_{2}\left|,...,\right|P_{n}-P_{n-1}|$$

### 6.2. Peak-to-Peak Cycle-to-Cycle-Jitter

To determine the larges difference between periods in a measured signal, the Jpp is used. As shown in Equation (6), it represents the difference between the shortest and the longest period of all samples in the measurement.

(6) Jpp=max(Jcc)−min(Jcc)

These periods do not have to be consecutive or adjacent to each other, but they can be. In that case the Jpp is equivalent to the maximum value of the Jcc.

### 6.3. Periodic Jitter

Jper is the deviation between the individual period and the ideal period. In some cases, there is no ideal reference clock available to compare against. In this case, it is common to use the arithmetic mean over all sampled periods as reference.

**Figure 11:**
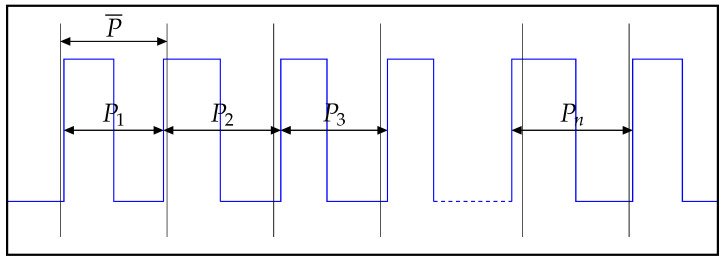
Periodic Jitter.

The signal in Figure 11 is described by its periods $P_{1}$ to $P_{n}$. To calculate the Jper for each period, the mean of all periods $\bar{P}$ is used.

(7) $$Jper=|\bar{P}-P_{1}\left|,\right|\bar{P}-P_{2}\left|,...,\right|\bar{P}-P_{n}|$$

The Periodic Jitter is calculated as shown in Equation (7). Jper is a numeric list, which contains the Jper value for each individual period within the measured signal. The symbol $\bar{P}$ is the mean over all individual periods $P_{1}$ to $P_{n}$.

### 6.4. Jitter Experiments

All forms or measurements of jitter described in the previous section were used to further investigate the timing behavior of the implemented Sensor-in-the-Loop approach. To do so, the timing experiments shown in Section 5 were extensively extended by the jitter experiments in this section.

#### Measurements

To compare the jitter of our implemented SiL approach, we measured the time for different sensor sampling methods. In these experiments, only the replay phase of the SiL interface was considered. The timing behavior of this phase is important, because it must be as similar as possible to the sampling of the real or live sensor data. The timing behavior of the record phase is not critical because the recorded sensor data have a time stamp and can be reconstructed reliably.

In Table 2, all evaluated firmware-dependent test scenarios of measuring are shown. All these scenarios were measured in both a compiler optimized version and an unoptimized version of the sensor firmware. For the optimized firmware, optimization level (-O3) was used and for the unoptimized version, (-O0) was used. Aside from these firmware-dependent scenarios, we also measured the timing behavior of the data ready interrupt of the hardware sensors used in the smart sensor. These measurements are not shown in the results later because they do not depend on the firmware running on the smart sensor. Furthermore, the jitter of these measurements is included in the measurement of scenario one and two.

Scenario one measures the time between two sensor samples (falling edge to falling edge) using the data ready interrupt of the accelerometer. The rising edge of the signal to measure is generated by the first instruction in the interrupt routine of the Microcontroller. The falling edge is generated by the next instruction after the sensor data are sampled successfully.

In the second scenario, the data are sampled from the gyroscope. The procedure is similar to the first scenario, except that the interrupt is invoked by the gyroscope and the data are sampled by the same.

The third scenario shows the time between two timer interrupts of the General Purpose Timer (GPT) build in the µC. This measurement is important to see how much of an influence the jitter of the GPT has on the following scenarios. All test scenarios from this point are using the GPT as interrupt source to start the sampling process.

In scenarios four and five, the sensor data are sampled from all three sensors available in the used smart sensor. The rising edge to measure is generate by the first instruction in the GPT interrupt. The falling edge is generated after the data of all three sensors is sampled successfully. In scenario four, the Sensor-in-the-Loop interface is used to sample the data of all sensors. In this case, the sensor data are prerecorded data that are injected by the presented Sensor-in-the-Loop approach. The accelerometer is sampled first, followed by the gyroscope and the magnetometer, all sensor one at a time. Scenario five is similar to scenario four but the SPI interface is used for the data sampling to obtain the data from the real hardware sensors.

In the last two scenarios, six and seven, just the accelerometer sampling is considered. Like in scenario four, in the sixth scenario the SiL interface is used to obtain the recorded sensor data from the accelerometer. The last scenario samples the accelerometer data over the SPI interface, also using the General-Purpose Timer interrupt as sampling trigger.

For all sensor interrupt dependent scenarios, the sensor devices were configured to produce a data ready interrupt with a frequency of 1000 Hz. The General-Purpose Timer of the Microcontroller was also configured to trigger one timer interrupt every 1 ms, this leads to a comparable sampling frequency of 1000 Hz for all evaluated scenarios.

## 7. Results

In this section, we will present the results of the timing analysis experiments done on our Sensor-in-the-Loop implementation. First, the results of the actual timing experiments are presented. The second subsection shows the results of the jitter experiments.

The names of the different experiments listed in the tables below are a composition of abbreviations. This makes it possible to have all information about the regarding experiment encoded in its name. In the following, we will give a brief description of these abbreviations. The *get* in the timing experiments and the *sample* in the jitter experiments marking scenarios getting or sampling sensor data. The *acc* is used for accelerometer and *all* or *9dof* for all three sensors accelerometer, magnetometer and gyroscope. The interface for sampling can be *spi* for serial peripheral interface or *RTT* for real-time transfer interface. There are two types of interrupts, *timer_int*, timer-based interrupt and *int_acc_int* or *int_gyr_int* for the data ready interrupt of the sensor device. The *record* means the recording feature of the SiL. The numbers describe the sampling frequencies during the experiment. A proper description of each experiment can be found in Section 5 or Section 6 in the experiments section.

### 7.1. Timing Analysis Results

The injection of sensor data described in Section 5 was done successfully. All injected sensor data could be processed by the firmware in the smart sensor and no data were lost during this process. The sensor ran in real time during the data injection and there was no issue with the program flow or timing behavior.

Table 3 shows the measured values for all analyzed scenarios in the debug configuration of the firmware. As one can see, with a mean of $1.15\times 10^{-4}$ s the sampling of one accelerometer sensor sample over the SPI interface takes more than four times as long as the sampling over the SiL interface, which is measured with $2.78\times 10^{-5}$ s.

When sampling more than one sensor, the duration of the sampling is usually *n* times longer than for a single sensor, were *n* is the number of sensors. Using the SPI interface this is true for the measured configuration.

In the case the data are sampled from the SiL interface, the timing behavior is a bit different. This is caused by the two-staged buffer structure depicted in Figure 7. If the data are sampled from the ring buffer or using the FIFO of the RTT interface, the timing behavior slightly differs. Even with this limitation the data streaming over SiL is with $7.02\times 10^{-5}\;$s still more than four times faster than the sampling of the hardware sensors.

To test the effect of the implemented two-staged buffer structure we executed a test case with different sampling rates on each sensor. This test was done to investigate if the buffers are filled unevenly. This could possibly have an influence on the timing behavior. The results of $4.5\times 10^{-5}\;$ s show a similar behavior to the sampling with an equal sampling frequency of all sensors.

The time for the pin toggle was measured with $4.7\times 10^{-6}\;$ s in the firmware compiled with the (-O0) option and $7.8\times 10^{-8}\;$s for the (-O3) optimized version. This time is included in the other results and must be subtracted from them to obtain absolute timing values.

The measurement of the data transmission from the sensor to the host was done to test the possibility to use the record function during real sensor operation. This feature has no influence on the timing behavior of the sensor data injection. For that, only the data flow from the host to the sensor is significant. With a time of $7.02\times 10^{-5}\;$s, the record feature is usable for most use cases to observe the sensor data in the running sensor in real time.

All measurements described for the firmware without optimization were also done with a firmware image with the (-O3) compiler optimization. In Table 4 all measured values for this test cases are listed. The optimization seems to have a bigger influence on the SPI communication code than it has on the SiL interface version. This leads to a bigger improvement on the timing for the SPI dependent test cases. However, the Sensor-in-the-Loop interface is still two times faster than sampling the data from the real sensors. The values measured in the optimized version are not compared in detail because (-O3) is usually not used in real-time applications. Furthermore, the SiL is a debugging and testing feature. In this kind of tasks mostly firmware compiled without optimization is used.

**Figure 12:**
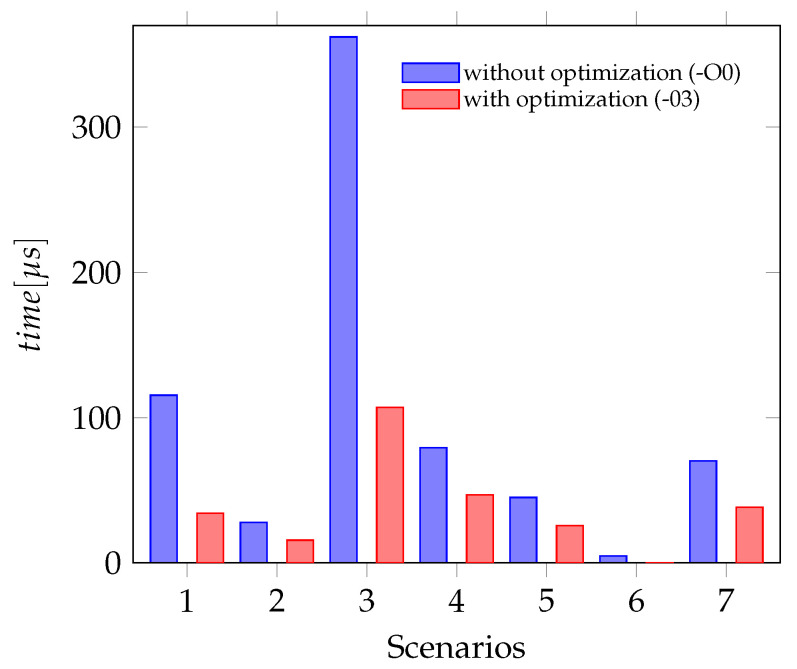
Timing behavior with and without compiler optimization (Scenario ID in Table 3 or Table 4).

Figure 12 shows that the SPI dependent scenarios (one and three) take more than four times longer to sample the sensor data. In this figure, scenario one must be compared to scenario two, both sample the data of the accelerometer only. The scenarios three and four sample the data of all three sensors. In both cases, the first scenario is sampling over SPI and the second is using the SiL interface.

### 7.2. Jitter Analysis Results

To achieve a better understanding of the timing behavior of our Sensor-in-the-Loop implementation. We performed a further investigation which focused on the jitter or timing difference between the individual sensor samples. The description of all different scenarios can be found in Section 6.

Table 5 shows the results of our jitter measurements for the Cycle-to-Cycle-Jitter. The measurements have been conducted with a firmware that was compiled without compiler optimizations. In this table, Jcc[s] is the maximal value in the list of all Jcc values for the measured signal. The largest jitter can be seen in the test scenarios of sensor sampling which uses the data ready interrupt of the hardware sensors. In these measurements, the jitter of the gyroscope is with $9.70\times 10^{-5}\;$s nearly three times worse than the jitter of the accelerometer. This diversion between two consecutive sampling times can be almost 10% of the sampling period for our use case with a sampling rate of 1000 Hz. The smallest jitter can be seen on the scenarios using the Sensor-in-the-Loop interface. If only one sensor is used the Jcc is only $2.80\times 10^{-6}$ s. The influence of the SPI interface is with $2.91\times 10^{-6}$ s for one sensor and $3.24\times 10^{-6}$ s for three sensors very small compared to the jitter caused by the hardware sensors itself.

The mean values for the Jcc are a lot smaller, usually by two orders of magnitude. If, from time to time, a larger difference between two adjacent sampling periods is tolerable for a use case, the mean values can be taken into account. As the Jpp describes the difference between the longest and the shortest period in the whole signal, it shows if there are significant timing differences over time in the signal. In that case, the Jpp was significantly larger than the maximum Jcc value. For our measurements, the Jpp values do not differ significantly from the Jcc values.

The compiler optimization of the firmware code can have a significant influence on the jitter. Table 6 shows the Jcc and Jpp jitter values for the test cases compiled with (-O3) optimization. The most considerable influence shows the jitter for the Sensor-in-the-Loop dependent sampling scenarios and the implementation of the timer interrupt routine. This has an influence on all timer dependent sampling scenarios. The least influence is shown in the scenarios one and two. The jitter of these two sampling scenarios mainly depends on the jitter of the hardware sensors and are not influenced by the compiler optimization.

**Figure 13:**
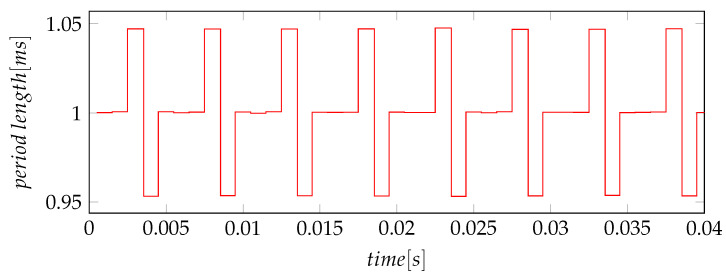
Periodicity of Gyroscope jitter.

A second type of jitter, which can be calculated from the measurements, is the Periodic Jitter, the definition and calculation can be seen in Section 6. This type describes the deviation of the current period from the ideal period or in the described case from the mean period. For the experiments done in this article, the Jper is smaller than the values for the Jcc. In some cases, for example for scenarios one and two, the Periodic Jitter is only half of the Cycle-to-Cycle-Jitter. This can be explained by having a closer look at the actual deltas in those signals. In Figure 13, the period lengths of the data ready interrupt of the gyroscope are plotted over time. In this signal, every long period is followed by a short one, this behavior leads do a larger value for the Jcc.

The tables, Table 7 and Table 8 contain the standard deviation of the period length. It shows that this value correlates with the jitter in some cases. However, taking a closer look, it shows that this does not hold for every case. For scenario one, for example, the standard deviation is with $5.54\times 10^{-7}\;$s two orders of magnitudes smaller than for scenario two with $2.97\times 10^{-5}\;$s. The Periodic-Jitters are with $1.68\times 10^{-5}\;$s and $4.82\times 10^{-5}\;$s in the equal order. Therefore, the standard deviation shows a trend in the period deviation but is not sufficient to assess the timing quality of the signal.

The influence of the compiler optimization on the Jper jitter values is similar to the Cycle-to-Cycle-Jitter values.

In Figure 13, the individual period lengths are shown over time. The pictured behavior explains why the Jcc values are twice as large as the Jper values. As said, a very short period is always followed by a very long period. The diagram also shows that the jitter of the gyroscope interrupt can be classified as deterministic Periodic Jitter.

**Figure 14:**
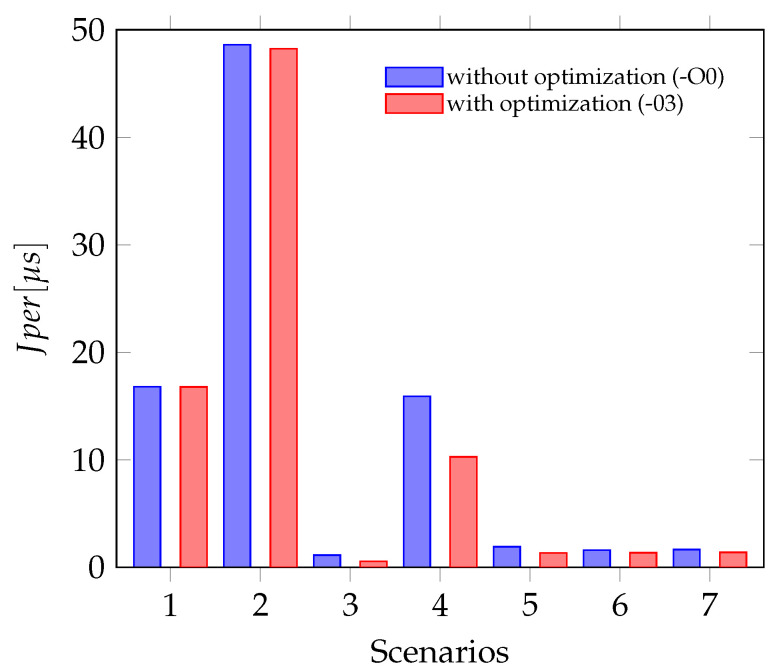
Periodic Jitter with and without optimization (Scenario numbering as in Table 2).

The Figure 14 shows the Perio dic Jitter for all seven scenarios measured. The test scenarios one and two, which include the jitter of the actual hardware sensors show a significant larger jitter than all other scenarios. The jitter in the scenarios where sensor data are sampled using the Sensor-in-the-Loop interface is similar but still less than the jitter caused by the accelerometer. Plotting the bars for optimized and unoptimized sensor firmware side by side, shows that the influence of the code optimization is not significant, especially if the sampling depends on a hardware sensor.

Since all examined jitters are lower for the scenarios using our SiL approach, a use of replayed sensor data will have little to no influence on the firmware and algorithms under test. All measured sampling jitters are less than five percent of the sampling frequency, which means an influence of the jitter on control algorithms is negligible [32].

## 8. Statistical Significance of Results

After presenting the actual results of our investigation on timing and jitter in Section 7, we will now examine the statistical significance of these results in this section. To analyze the influence of different parameters on our results, a statistical analysis of the results has been conducted.

### 8.1. Statistical Analysis Methods

The analysis of the statistical significance of the results has been done using two methodologies which are described below.

#### 8.1.1. Gage R&R

The first methodology used is the Gage R&R analysis [33] which is commonly used for measurement system analysis to assess repeatability and reproducibility of a measurement system. Said method applies an analysis of variance to estimate the influence of three independent factors on the overall results of the measurements. The traditional Gage R&R analysis is done with the three factors *tested part*, *testing person* and *test repetitions* [34]. In the case of the work at hand, the analysis determines the influence of the *method*, the *scenario* and the *repetition* of the measurement on dependent variable, the *jitter*.

For the calculation the variance, range and mean are calculated grouped by each of the independent variables. The sum of squares error is calculated within each group compared to the group mean and between all groups compared to the mean of all results. These values are then put into relation to determine the influence of each group on the mean and variance of the overall result. The equations and calculation rules can be found in [35].

#### 8.1.2. Confidence Interval

The second methodology is the confidence interval [35]. With the confidence interval it is possible to estimate a range in which the true value of an unknown parameter from a set of measured values is for a given probability. The probability is previously defined, most of the time a probability of 95% is used. The calculation of the confidence interval is shown in Equation (8).

(8) $$CI=\bar{X}\pm Z^{*}\cdot \frac{\sigma }{\sqrt{n}}$$

$\bar{X}$ is the mean value of the measured values, σ is the standard deviation of the measured values and *n* is the number of samples. The value for $Z^{*}$ can be taken from a table for a given number of samples and required probability. It is important to note that for a sample size of more than 100, the table for a standard normal distribution (or Z-Distribution) can be used. For a sample size lower than 100, a table using the Student’s t-distribution is required. The ± indicates that the same equation is used to calculate the upper and lower bounds of the confidence interval. To show that multiple groups of measurements (e.g., in the case of the work at hand by scenario) can be distinguished, the confidence interval for each group should be narrow and none of the intervals overlap.

### 8.2. Results of the Analyses

In this section, the results of the two statistical analyses are presented for the Cycle-to-Cycle-Jitter.

We performed the Gage R&R analysis for both test cases separately, one for the firmware image compiled without optimization and a separate analysis for the compiler optimized version. To obtain a statistically significant result and proof this by doing such a significance test, we generate a common amount of test data. For that, we executed our tests with three different sensors. On these three sensors, we tested our seven different scenarios in the optimized and non-optimized versions. Each of these tests was done three times. The data rate for these test cases was 1000 Hz and each run was done for 10 s. This leads to an overall amount of 1,260,000 data points for both Gage Repeatability analyses.

The results of the statistical analysis for the test cases without compiler optimization, can be seen in Table 9. The row *Contribution [%]* shows that the method, i.e., the scenario, has, by far, the largest influence on the total variation. The remaining two parameters only contribute marginally to the overall result. The influence of the part or the different sensors used is close to zero. The repetition of the experiment also has close to zero influence on the overall result.

As said before, we also did the same experiments with a compiler optimized firmware version. The results for the optimized firmware are depicted in Table 10. The statistical analysis shows nearly the same results as the unoptimized one. In this case, the scenario has also over 99% influence on the overall result. The values for the part and the repetitions are close to zero.

The Gage Repeatability analysis shows that the results for the Cycle-to-Cycle-Jitter values are statistically significant. The results of the analysis and the small values in for the variance of the data shows that the experiments are valid to determine the timing behavior and jitter of the Sensor-in-the-Loop implementation. Furthermore, because of the little influence of the repeatability on the overall result, we conclude that the timing and the jitters are not data-dependent.

**Figure 15:**
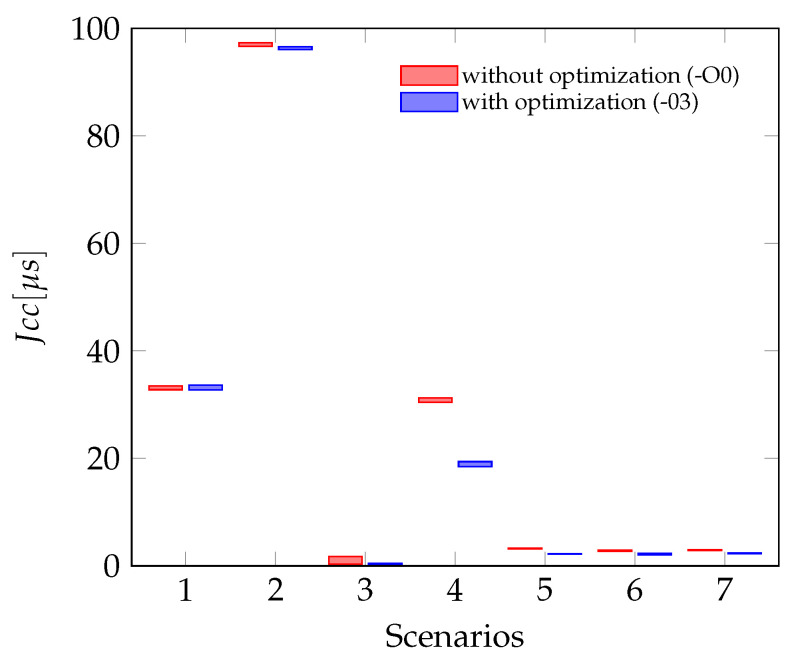
Confidence intervals for Jcc with and without code optimization.

To have a second gage for the statistical significance of the timing and jitter results, we calculated the confidence intervals for the Jcc results for all measured scenarios. These confidence intervals are shown in Figure 15. For all scenarios, the confidence intervals are relatively small compared to the calculated mean of the Jcc values. Furthermore, there is no significant overlap of the individual confidence intervals of each scenario. The analyses of the confidence intervals substantiate the results from the Gage R&R analyses that our timing and jitter experiments deliver statistically significant results.

## 9. Discussion

Due to the higher integration levels of microprocessors in recent years, the market of sensors changed from using simple dedicated sensor, to using high integrated smart sensors. This change enables sensor developer to implement sensor tasks such as processing sensor data or more complex algorithms such as gesture recognition directly on the sensor. Firmware development on smart sensors can be quite challenging, especially when it is necessary to test software parts in different test runs. With state-of-the-art methods is not possible to do repeatable or even reproducible test, directly on the sensor hardware.

In this article, we presented our approach for a Sensor-in-the-Loop architecture. This approach can significantly improve the development process of firmware for smart inertial sensors. It allows the developer to test the firmware in a repeatable and reproducible way. This is possible by injecting previously recorded or artificially generated sensor data into the running sensor firmware in real time.

We identified the criteria that must be met to implement such a SiL architecture, so that it will work in real time. Following these criteria, we designed an example implementation of our approach using the BMF055, a state-of-the-art smart inertial sensor by Bosch Sensortec. The evaluation of this implementation was done using different sensor configurations. These configurations used one or multiple hardware sensors at a time. Even configurations with multiple sensors and different sampling rates were tested.

In comparison to simulation-based approaches described in [13] or [12], we work on the real hardware of the smart sensor. This can be a limitation, if the real hardware is not available yet. If the sensor itself is available, it can be a benefit because it is possible to observe the behavior immediately at runtime. Furthermore, extra functional properties such as power dissipation can be observed during the development. Compared to other approaches using the real sensor hardware [15,16], we can use the same sensor data in different test runs. This makes it possible to obtain repeatable and reproducible results. To use this approach, it is mandatory to modify the sensor firmware in preparation of the usage, this is an extra step in development of the sensor firmware. The presented implementation can reproduce the timing behavior of using real sensor devices quite well. However, it is not possible to reproduce the behavior in timing and jitter perfect, but it fits most use cases.

The evaluation experiments we did on our example implementation are dived into two different main experiments.:

At first, we investigated the overall performance of our implementation, this was done in Section 5. To do this, we assessed whether it is possible to record and inject different sensor configurations in real time with repeatable results. The tests were done with a maximum of three sensors, each of the sensors consists of three sensor axes. The results showed that it is possible to inject the sensor data with a sampling rate of 1600 Hz and obtain repeatable and reproducible results in real time using our proposed approach.

The second experiments focused on the jitter in the sampling process. We measured and compared different kinds of jitter for the sampling of the actual hardware sensors and using our Sensor-in-the-Loop architecture. Furthermore, we measured the jitter for different hardware configurations, e.g., timer-based sampling or interrupt-based sampling. The results show that the sampling jitter is different for sampling actual hardware sensors and sampling using our SiL approach. That means that our implementation cannot reproduce the timing behavior perfectly. Considering this, the jitter for the SiL implantation has the same order but remains smaller than the jitter for the actual sensor devices, we conclude that this implementation reproduces the timing behavior close enough to use it for most use cases. According to [32] the measured jitters are in a negligible range. So, we can assume that the jitter has no influence on the implemented algorithms, no matter if using real or artificial sensor data.

To prove the statistical significance of our experiments, we used two different statistical methods. We used the Gage Repeatability analysis to investigate the influence of the different parameters on our results. Using this, we showed that the results are data independent and are not influenced using different sensors of the same kind. The Gage R&R test showed that the actual measured scenario has more than 99% influence on the result. The small variance in the analysis results proof the statistical significance of our test data. To substantiate the Gage R&R analysis, we also calculated the confidence interval of our test data. The small confidence interval in comparison to the calculated mean value and the small overlap of the separate intervals also speaks for statistically significant data. We performed the statistically tests only for Cycle-to-Cycle-Jitter data as all other jitter and timing calculations are statistically connected.

## 10. Conclusions

In this article, we presented our Sensor-in-the-Loop approach, which can be a great aid for developer of smart sensor firmware. It can be used to inject prerecorded or artificial sensor data directly into the smart sensor in real time. This enables the developer to do repeatable and even reproducible test on the real sensor hardware during the development process.

We showed that our approach can handle sensor data of three 3-DOF Sensors with a sampling rate of 1.6 kHz in real time. Furthermore, we investigated the influence of our implementation on the timing behavior and jitter. The influence on the timing and jitter is negligible, this enables our approach to be used on a wide range of use cases.

The timing analyses were confirmed by two statistical significance analysis methods. The conducted Gage Repeatability analysis as well as the confidence intervals support the correctness of our test results.

In future studies we are planing to use our approach for investigation on extra functional properties in real world smart sensor application.

## Figures and Tables

**Table 1:** Sensor-in-the-Loop compared to state-of-the-art technologies.

Approach	Considers	Execution	Reproducible	Extra Functional Properties
Sensor-in-the-Loop	Whole system	fast	yes	yes
Functional Simulation	Only data	fast	yes	no
Virtual Prototype	Parts of the system	slow	yes	partially
Hardware Prototype	Whole system	slow	no	yes

**Table 2:** Measured test scenarios of sampling sensor data.

Scenario ID	Scenario
1	int_acc_int_spi_sample
2	int_gyr_int_spi_sample
3	timer_int
4	timer_int_9dof_SiL_sample
5	timer_int_9dof_spi_sample
6	timer_int_acc_SiL_sample
7	timer_int_acc_spi_sample

**Table 3:** Measured timing behavior without optimization (-O0).

ID	Name	Max [s]	Min [s]	Mean [s]	Std Dev
1	get_acc_spi_api_1600	1.17 × 10^−4^	1.15 × 10^−4^	1.15 × 10^−4^	1.34 × 10^−7^
2	get_acc_RTT_1600	2.93 × 10^−5^	2.70 × 10^−5^	2.78 × 10^−5^	3.21 × 10^−7^
3	get_all_spi_api_1600	3.64 × 10^−4^	3.62 × 10^−4^	3.62 × 10^−4^	2.51 × 10^−7^
4	get_all_RTT_1600	9.47 × 10^−5^	6.36 × 10^−5^	7.92 × 10^−5^	1.86 × 10^−6^
5	get_all_RTT_800_1600_100	8.64 × 10^−5^	2.85 × 10^−5^	4.50 × 10^−5^	1.20 × 10^−5^
6	pin_toggle_reference	6.00 × 10^−6^	4.73 × 10^−6^	4.74 × 10^−6^	1.98 × 10^−8^
7	ecord_Acc_1600	7.30 × 10^−5^	6.95 × 10^−5^	7.02 × 10^−5^	2.78 × 10^−7^

**Table 4:** Measured timing behavior with optimization (-O3).

ID	Name	Max [s]	Min [s]	Mean [s]	Std Dev
1	get_acc_spi_api_1600	3.52 × 10^−5^	3.41 × 10^−5^	3.42 × 10^−5^	5.03 × 10^−8^
2	get_acc_RTT_1600	1.67 × 10^−5^	1.49 × 10^−5^	1.56 × 10^−5^	2.47 × 10^−7^
3	get_all_spi_api_1600	1.08 × 10^−4^	1.07 × 10^−4^	1.07 × 10^−4^	9.40 × 10^−8^
4	get_all_RTT_1600	5.70 × 10^−5^	3.75 × 10^−5^	4.68 × 10^−5^	1.06 × 10^−6^
5	get_all_RTT_800_1600_100	5.12 × 10^−5^	1.51 × 10^−5^	2.56 × 10^−5^	7.51 × 10^−6^
6	pin_toggle_reference	8.00 × 10^−8^	7.80 × 10^−8^	7.87 × 10^−8^	9.43 × 10^−10^
7	record_Acc_1600	4.00 × 10^−5^	3.70 × 10^−5^	3.82 × 10^−5^	4.20 × 10^−7^

**Table 5:** Cycle-to-Cycle-Jitter without optimization (-O0).

Name	Jcc [s]	Jcc on Signal [%]	Mean Jcc [s]	Jpp	Jpp on Signal [%]
int_acc_int_spi_sample	3.31 × 10^−5^	3.35	3.43 × 10^−7^	3.33 × 10^−5^	3.37
int_gyr_int_spi_sample	9.70 × 10^−5^	9.70	3.77 × 10^−5^	9.71 × 10^−5^	9.70
timer_int	1.01 × 10^−6^	0.10	1.90 × 10^−7^	1.70 × 10^−6^	0.17
timer_int_9dof_SiL_sample	3.08 × 10^−5^	3.04	6.02 × 10^−7^	3.14 × 10^−5^	3.10
timer_int_9dof_spi_sample	3.24 × 10^−6^	0.32	3.57 × 10^−7^	3.70 × 10^−6^	0.36
timer_int_acc_SiL_sample	2.80 × 10^−6^	0.28	1.64 × 10^−7^	3.09 × 10^−6^	0.30
timer_int_acc_spi_sample	2.91 × 10^−6^	0.29	1.92 × 10^−7^	3.21 × 10^−6^	0.32

**Table 6:** Cycle-to-Cycle-Jitter with optimization (-O3).

Name	Jcc [s]	Jcc on Signal [%]	Mean Jcc [s]	Jpp	Jpp on Signal [%]
int_acc_int_spi_sample	3.32 × 10^−5^	3.36	2.69 × 10^−7^	3.33 × 10^−5^	3.37
int_gyr_int_spi_sample	9.63 × 10^−5^	9.63	3.77 × 10^−5^	9.64 × 10^−5^	9.64
timer_int	4.01 × 10^−7^	0.04	7.11 × 10^−8^	1.04 × 10^−6^	0.10
timer_int_9dof_SiL_sample	1.89 × 10^−5^	1.87	3.44 × 10^−7^	2.00 × 10^−5^	1.98
timer_int_9dof_spi_sample	2.23 × 10^−6^	0.22	1.29 × 10^−7^	2.60 × 10^−6^	0.26
timer_int_acc_SiL_sample	2.20 × 10^−6^	0.22	1.34 × 10^−7^	2.56 × 10^−6^	0.25
timer_int_acc_spi_sample	2.35 × 10^−6^	0.22	1.31 × 10^−7^	2.64 × 10^−6^	0.26

**Table 7:** Periodic Jitter without optimization (-O0).

Name	Std	Jper [s]	Mean Jper [s]	Jper on Signal [%]
int_acc_int_spi_sample	5.42 × 10^−7^	1.68 × 10^−5^	2.24 × 10^−7^	1.70
int_gyr_int_spi_sample	2.97 × 10^−5^	4.86 × 10^−5^	1.89 × 10^−5^	4.86
timer_int	2.96 × 10^−7^	1.12 × 10^−6^	1.96 × 10^−7^	0.11
timer_int_9dof_SiL_sample	1.54 × 10^−6^	1.59 × 10^−5^	3.70 × 10^−7^	1.57
timer_int_9dof_spi_sample	3.90 × 10^−7^	1.90 × 10^−6^	2.40 × 10^−7^	0.19
timer_int_acc_SiL_sample	2.05 × 10^−7^	1.60 × 10^−6^	1.44 × 10^−7^	0.16
timer_int_acc_spi_sample	2.46 × 10^−7^	1.64 × 10^−6^	1.57 × 10^−7^	0.16

**Table 8:** Periodic Jitter with optimization (-O3).

Name	Std	Jper [s]	Mean Jper [s]	Jper on Signal [%]
int_acc_int_spi_sample	4.89 × 10^−7^	1.68 × 10^−5^	1.88 × 10^−7^	1.70
int_gyr_int_spi_sample	2.97 × 10^−5^	4.82 × 10^−5^	1.89 × 10^−5^	4.82
timer_int	1.40 × 10^−7^	5.60 × 10^−7^	1.13 × 10^−7^	0.06
timer_int_9dof_SiL_sample	8.98 × 10^−7^	1.03 × 10^−5^	2.47 × 10^−7^	1.01
timer_int_9dof_spi_sample	1.96 × 10^−7^	1.34 × 10^−6^	1.35 × 10^−7^	0.13
timer_int_acc_SiL_sample	1.75 × 10^−7^	1.35 × 10^−6^	1.31 × 10^−7^	0.13
timer_int_acc_spi_sample	1.75 × 10^−7^	1.38 × 10^−6^	1.31 × 10^−7^	0.14

**Table 9:** Gage R&R significance result for Jcc without optimization.

	Part-to-Part	Method	Repeatability	TOTAL Variation
Variance (VarComp)	1.04 × 10^−14^	1.22 × 10^−9^	7.04 × 10^−14^	1.22 × 10^−9^
Contribution [%]	0.001	99.980	0.010	100.000
Standard Deviation	1.00 × 10^−7^	3.49 × 10^−5^	2.65 × 10^−7^	3.49 × 10^−5^
Study Variation [%]	0.290	99.990	0.760	100.00

**Table 10:** Gage R&R significance result for Jcc with optimization.

	Part-to-Part	Method	Repeatability	TOTAL Variation
Variance (VarComp)	0.00 × 10^−0^	1.22 × 10^−9^	7.68 × 10^−14^	1.22 × 10^−9^
Contribution [%]	0.000	99.990	0.010	100.000
Standard Deviation	0.00 × 10^−0^	3.49 × 10^−5^	2.77 × 10^−7^	3.49 × 10^−5^
Study Variation [%]	0.000	99.990	0.790	100.00

## Abbreviations

µC	Microcontroller
SoM	System on Module
SiP	System in Package
SoC	System On Chip
SiL	Sensor-in-the-Loop
MEMS	Micro-Electro-Mechanical Systems
ADC	Analog to Digital Converter
SPI	Serial Peripheral Interface
I${}^{2}$C	Inter-Integrated Circuit
AP	Application Processor
Jcc	Cycle-to-Cycle-Jitter
Jper	Periodic Jitter
Jpp	peak-to-peak Cycle-to-Cycle-Jitter
UART	Universal Asynchronous Receiver Transmitter
GPT	General-Purpose Timer
RTT	Real-Time Transfer
SWD	Serial Wire Debug Interface
Gage R&R	Gage Repeatability and Reproducibility
HiL	Hardware-in-the-Loop
SPU	Sensor-Processing Unit
VCI	View and Control Interface
GUI	Graphical User Interface
DOF	Degrees of Freedom
UAV	Unmanned Aerial Vehicle
CSV	Comma Separated Values
FIFO	First In First Out
GPIO	General-Purpose Input Output
TCP	Transmission Control Protocol
ROM	Read Only Memory
IDE	Integrated Development Environment
API	Application Programming Interface
